# How the Lack of Chitosan Characterization Precludes Implementation of the Safe-by-Design Concept

**DOI:** 10.3389/fbioe.2020.00165

**Published:** 2020-03-10

**Authors:** Cíntia Marques, Claudia Som, Mélanie Schmutz, Olga Borges, Gerrit Borchard

**Affiliations:** ^1^Institute of Pharmaceutical Sciences of Western Switzerland, University of Geneva, Geneva, Switzerland; ^2^Faculty of Pharmacy, University of Coimbra, Coimbra, Portugal; ^3^Empa, Swiss Federal Laboratories for Materials Science and Technology, Technology and Society Laboratory, St. Gallen, Switzerland; ^4^Center for Neuroscience and Cell Biology, University of Coimbra, Coimbra, Portugal

**Keywords:** safe by design, polymeric drug carriers, chitosan, insulin, protein drug delivery

## Abstract

Efficacy and safety of nanomedicines based on polymeric (bio)materials will benefit from a rational implementation of a Safe-by-Design (SbD) approach throughout their development. In order to achieve this goal, however, a standardization of preparation and characterization methods and their accurate reporting is needed. Focusing on the example of chitosan, a biopolymer derived from chitin and frequently used in drug and vaccine delivery vector preparation, this review discusses the challenges still to be met and overcome prior to a successful implementation of the SbD approach to the preparation of chitosan-based protein drug delivery systems.

## Introduction

Nanoparticles (NPs) have been extensively investigated as delivery systems for targeted drug delivery, controlled drug release, *in vivo* imaging, diagnostics, and medical devices. These systems may offer more convenient routes of administration, decrease drug toxicity, and potentially reduce healthcare costs (Vasile, 2019). However, despite numerous publications on nanoparticulate drug carrier systems (“nanomedicines”), the extent of their translation into clinical application has been unsatisfactory (Hua et al., 2018; Rosenblum et al., 2018). The first generation of these nanomedicines passed regulatory approval by meeting standards in place for “conventional” drugs of low molecular weight. However, with regard to the complexity of nanomedicines, these standards were reviewed and partially replaced by nano-specific critical quality attributes (CQAs) that need to be reported in order to confirm quality, safety, and efficacy of NPs (Gaspar, 2007; U. S. Food and Drug Administration, 2017). Quality control assays for nanomaterial characterization, the need of establishing specialized toxicology studies for nanomedicines, and the lack of suitable standards and dedicated regulatory guidelines are a few examples of the challenges to their development and effective clinical translation (Hua et al., 2018).

The research community is working to establish protocols for nanomaterial characterization (Brown et al., 2010). The Nanotechnology Regulatory Science Research Plan, established by the Food and Drug Administration (FDA), addresses five major criteria, namely, physicochemical characterization, pre-clinical models, risk characterization, risk assessment, and risk communication (Rosenblum et al., 2018). In this regard, the Nanotechnology Characterization Laboratory (US NCL) was founded, focusing on the characterization of nanomedicines for cancer therapy. In Europe, the European Nanomedicine Characterization Laboratory (EU-NCL) was created as a multi-national organization within the H2020 framework. EU-NCL focuses on the pre-clinical characterization of nanomaterials in order to accelerate their development toward the approval by the regulatory agencies (European Nanomedicine Characterization Laboratory, 2019a). Moreover, in the European Union, other projects such as NANoREG, NANoREG II, ProSafe, and NanoDefine have also explored the standardization of nanomaterial characterization, and the development of better prediction models, such as the application of the Safe-by-Design (SbD) approach to nanosystems (Kraegeloh et al., 2018).

The principle behind SbD includes the safety assessment of nanomedicines as early as possible in their innovation process and throughout their lifecycle by designing out the physicochemical properties with an adverse effect on human health and the environment (Bottero et al., 2017; Soeteman-Hernandez et al., 2019). Several concepts of SbD have arisen from the European projects mentioned above. For example, the NANoREG project describes three pillars: safe product by design, safe use of products and safe industrial production. In addition, according to NANoREG II, the SbD concept aims at the development of functional and safer nanomaterials, safer processes as well as safer products. In general, the application of this concept requires the examination of which physicochemical properties render a nanomaterial safe, means to implement this knowledge into industrial innovation processes, and information exchange between stakeholders. The SbD concept can be implemented to design nanomaterials with an optimal balance between functionality and risk, based on relevant physicochemical parameters (Kraegeloh et al., 2018).

The European project GoNanoBioMat created a SbD approach to support industries, particularly small and medium-size enterprises (SMEs) to identify risks and uncertainties early in the research and development phase, support safe production and handling, and deliver safe products. The SbD approach is applied to polymeric nanobiomaterials for drug delivery and it focuses on safe nanobiomaterials, safe production and safe storage and transport (Som et al., 2019).

Particularly, one goal of GoNanoBioMat was to establish the characteristics of different types of chitosan nanoparticles (Chit NPs), to establish a correlation between the physicochemical properties of this biopolymer and its immunostimulatory activity and, finally, to establish a guideline to select the most suitable chitosan polymer according to its purpose, allowing an SbD approach. To address these points, an extensive literature search was initiated and will be presented in this report.

Chitosan, the deacetylated form of chitin, is a biopolymer investigated for the preparation of particles as vectors for drug delivery. Chitosan nanoparticles are under investigation for a wide variety of biomedical applications, due to the polysaccharide’s exceptional versatility (Koppolu et al., 2014). One of the major applications of chitosan is the preparation of medical micro- and nano-particles. Nanoparticles of natural polymers are a promising approach for drug delivery due to their biocompatibility and biodegradability, as well as for their ability to provide a controlled drug release profile (Erel et al., 2016). Even though chitosan is one of the most studied biopolymers, there is no standardization as far as its properties and the resulting biological activity are concerned.

The goal of this review was to understand whether it is possible to identify physicochemical properties of chitosan that are correlated to its biological effects. To this end, supportive information on protocols used to prepare chitosan NPs encapsulating insulin (Chit-Ins NPs) as a model protein drug were collected. Protocol details and Chit-Ins NPs characterization data were compared. Literature was also examined for available information on the immunotoxicological response to Chit-Ins NPs administration. Finally, the report summarizes the current state of the art, identifies the challenges in applying the SbD concept to the bionanomaterial chitosan and establishes future perspectives on Chit NPs characterization.

## Methods

A literature search was performed through PubMed and Science Direct using as Medical Subject Headings (MeSH) keywords chitosan, immune activity, gelation, insulin, encapsulation, and adjuvant. We focused on ionotropic gelation, using tripolyphosphate (TPP) as crosslinker because it is the most used process to prepare Chit NPs. Insulin was chosen as a model for protein encapsulation into these nanoparticles.

## Chitosan: Potential and Versatility

**FIGURE 1:**
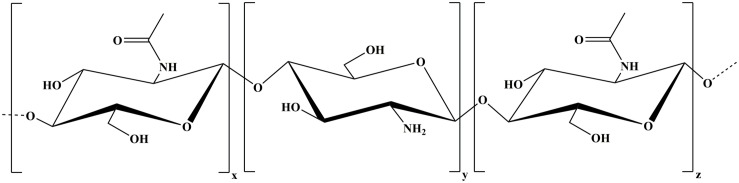
Chitosan composed of randomly distributed deacetylated unit (y, z) and acetylated unit (x).

Chitosan is the partially deacetylated form of chitin – a poly (D-glucosamine) – and comprises a wide range of linear polymers differing in polymer length and deacetylation degree. The polymer is composed of randomly distributed β-(1→4)-linked D-glucosamine (deacetylated unit) and N-acetyl-D-glucosamine (acetylated unit) (Figure 1) and it appears in the market with different purity degrees (Primex, 2019). Chitin is a natural biopolymer extracted from the exoskeleton of crustaceans (shrimp, crabs, lobsters, etc.) and from the cell walls of fungi or yeast (Illum, 1998; Mukhopadhyay et al., 2013; Vasiliev, 2015; Bugnicourt and Ladavière, 2016; Jafary Omid et al., 2018; Primex, 2019).

In fact, chitosan is one of the most studied biopolymers. This polysaccharide is exceptionally versatile as it can be used in solutions, suspensions, hydrogels and/or micro- and nanoparticles. Moreover, it is possible to proceed to its chemical functionalization through its amino and hydroxyl groups, and/or by conjugation of peptides and other molecules to the polymer backbone. This allows for the modification of physicochemical properties and/or the introduction of desirable characteristics, further broadening chitosan potential applications (Sreekumar et al., 2018).

Chitosan is well known for its inherent biological properties, namely biocompatibility (Hirano et al., 1990), non-toxicity (Hu et al., 2011; Pradines et al., 2015), antimicrobial activity (Zheng and Zhu, 2003; Qin et al., 2006; Cerchiara et al., 2015), plant strengthening (Choudhary et al., 2017), hydrating ability (Cerchiara et al., 2015), gel and film forming (Shan et al., 2010; Nie et al., 2016), mucoadhesive properties (Cerchiara et al., 2015; Patel et al., 2015), immunostimulant activity (Nishimura et al., 1985; Scherliess et al., 2013), hemocompatibility (Malette et al., 1983; Lee et al., 1995; Zhao et al., 2011), and biodegradability (Lee et al., 1995; Patel et al., 2015).

This polymer is one of the most widely used for biomedical applications. Actually, chitosan has been under investigation for drug and vaccine delivery (Borges et al., 2008; Esmaeili et al., 2010; Jafary Omid et al., 2018; Soares et al., 2018; Bento et al., 2019), gene delivery (Thanou et al., 2002), surgical sutures (Muzzarelli et al., 1993; Altinel et al., 2018), rebuilding of bone (Lee et al., 2009), corneal contact lenses (Silva et al., 2016), dental implants (Yokoyama et al., 2002), wound healing (Mizuno et al., 2003), antimicrobial applications (Dai et al., 2009), and tissue engineering (Madihally and Matthew, 1999; Kanimozhi et al., 2016). Moreover, chitosan has been used as a dietary supplement in preparations for treatment of obesity and hypercholesterolemia (Bokura and Kobayashi, 2003; Zhang et al., 2008) and also in medical devices for the treatment and control of bleeding (Millner et al., 2009). The polysaccharide is classified by FDA as Generally Recognized As Safe (GRAS) for food (Nutrition Center for Food Safety Applied, 2019a, b). The polymer description was first introduced into the European Pharmacopeia 6.0 and the 29th edition of the United States Pharmacopeia (USP) 34-NF. Monographs contain the assays and establish limits to be observed when the polymer is used as a pharmaceutical excipient (Council of Europe, 2019). Currently the efficacy of chitosan nanoparticles in the treatment of postoperative pain and antibacterial activity against *Enterococcus faecalis* in infected root canals is being studied in a phase 2 clinical trial (U. S National Library of Medicine, 2018).

## Challenges for Safe-By-Design of Chit-NPs

### Characterization of Chitosan Is Not Standardized

Despite the large number of papers about chitosan, reproducibility of the reported results is often an issue (Nasti et al., 2009). As mentioned above, chitosan is a family of polymers, which differ in their degree of deacetylation (DD), molecular weight (MW) and purity. The different characteristics can be correlated with the diversity of physicochemical properties and diverse biological activities of the polysaccharide. As a matter of fact, these structural characteristics are dependent on the source of chitin, its extraction, and the deacetylation method (Bellich et al., 2016), whose correlations with chitosan biological properties has been reviewed elsewhere (Younes and Rinaudo, 2015).

As illustrated in Table 1, chitosan basic characterization is neglected in many papers making it difficult to critically comment on conflicting experimental results (Vasiliev, 2015; Bellich et al., 2016). Even when the MW is provided, there is often an ambiguous classification. For example, Mehrabi et al. (2018) classify chitosan into high molecular weight (HMW) at the range of 700–1,000 kDa, low molecular weight (LMW) when less than 150 kDa, and medium molecular weight (MMW) between low and high molecular weight. On the other hand, Vila et al. (2004) mention chitosan of 23 and 38 kDa as LMW and chitosan of 70 kDa as HMW.

**TABLE 1:** Summary of Chit-Ins NPs production protocols by ionotropic-gelation method.

			Preparation method					NP characterization					
				
System	Insulin, source	Chitosan, source	Chitosan solution	Insulin solution		TPP solution	Final pH	Size	Zeta Potential	Insulin AE%	Toxicity assay	Anti-insulin IgG	References
Chit NPs	Porcine pancreas insulin, Sigma	186 kDa; 85% DDA Aldrich Chemicals	8 mL chitosan 2 mg/mL	Insulin 31.65 μg/mL to 235.25	1 mL premixed with TPP or	4 mL TPP 1 mg/mL	pH 2.8 to 6.1	237 nm ±53 nm to 325 nm ±45 nm	–	2–85%	–	–	Ma et al., 2002
Chit NPs	Insulin 27.6 I.U/mg, Xuzhou biochemical plant	? kDa 88.9% DDA; viscosity 45 mPa.s Shenyang	4 mL chitosan 2.6 mg/mL	Concen- tration? mins/mChit = 0.1	Solution Premixed with TPP solution	? mL TPP 0.45 mg/mL	–	265.3 nm ±34.1 nm	+40.71 mV ±0.69 mV	88.6% ± 2.4%	–	–	Pan et al., 2002
Chit NPs	Porcine pancreas insulin 27.8 USP/mg, Sigma Chemicals	186 kDa; 85% DDA Aldrich Chemical, Milwaukee	8 mL chitosan 2 mg/mL	Insulin 2 mg/mL in 0.01 M HCl	1 mL premixed with TPP solution	4 mL TPP 1 mg/mL in 0.05 M NaOH	pH 5.3	269 nm ± 7 nm	+34.9 mV ± 0.9 mV	38.5% ± 1.5%	–	–	Ma et al., 2005
						4 mL TPP 1 mg/mL in 0.075 M NaOH	pH 6.1	339 nm ± 8 nm	+21.8 mV ± 0.6 mV	78.5% ± 2.3%	–	–	
Chit NPs	Human insulin Novolin R^®^, 100 IU/mL	Low viscosity chitosan ? kDa; DDA ?	5 mL chitosan 4 mg/mL	Insulin solution 4.6 mg/Ml	Premixed with TPP solution	2 ml TPP 1 mg/mL	pH 6.1	312.8 nm PDI 0.48	+23 mV ± 2 mV	69.37% ± 4.71%	–	–	Azevedo et al., 2011
Chit NPs	Bovine pancreas insulin (27 USP/mg) Sigma-Aldrich, United States	200 kDa DDA ? Sigma-Aldrich, United States	? mL chitosan 2 mg/mL	Insulin solution 0.5 mg/ml	Premixed with TPP solution	? ml TPP 0.5 mg/mL	pH 5.5	215 nm PDI 0.16	+20.7 mV ± 0.7 mV	49.43% ± 0.44%	–	–	Makhlof et al., 2011
Chit NPs	Crystalline recombinant human insulin Novo Nordisk, Denmark	LMWC; 98% DDA; viscosity 22 cP	10 mL chitosan 1 mg/mL or 3 mg/mL	Insulin 0.5 mg/mL and 1 mg/mL (concentration in TPP)	Premixe d with TPP solution	? ml TPP solution 1 mg/mL and 3 mg/mL	–	261 nm PDI 0.4 or 419 nm PDI 0.45	+27.2 mV or +48.4 mV	61.61% ± 4.52% or 61.88% ± 5.59%	–	–	Kouchak et al., 2012
		MMWC; 92% DDA; viscosity 715 cP Primex, Iceland	10 mL; chitosan 0.5 mg/mL or 1 mg/mL					132 nm PDI 0.28 or 343 nm PDI 0.49	+25.1 mV or +39.3 mV	70.89% ± 3.32% or 70.59% ± 1.70%	–	–	
		HMWC; 96% DDA; viscosity 1234 cP Primex, Iceland	10 mL; chitosan 0.5 mg/mL or 1 mg/mL					112 nm PDI 0.27 or 160 nm PDI 0.28	+27.5 mV or +29.0 mV	53.50% ± 2.61% or 53.73% ± 2.29%	–	–	
Chit NPs	Zinc-free human insulin	150 kDa; 87% DDA; viscosity 2.37 dL/g Sigma-Aldrich, Missouri	? mL chitosan 2.5 mg/mL (in acetic acid)	4 mg/mL insulin solution	Premixed with TPP solution	? mL TPP 0.25 mg/mL	pH 5.5	330 nm ± 36 nm	+30 mV ± 4 mV	55% ± 8%	No death or inflammatory response (CAM assay in fertilized chicken eggs)	–	Rampino et al., 2013
Chit NPs	Insulin 27.5 IU/mg Jiangsu Wangbang Bio- Technology	400 kDa; DDA? Haixin Biological Product	? mL chitosan 50 mg (in acetic acid)	4 mg insulin solution in NaOH	Premixed with chitosan solution	3 mL TPP solution 0.5 mg/mL	pH 3	91.28 nm ± 7.9 nm to 220.2 nm ± 9.5 nm	+14.4 mV ± 2.9 mV	93.1%	–	–	Zhao et al., 2014
Chit NPs into transdermal patch	Pure insulin powder Sigma- Aldrich	LMWC; DDA? Sigma-Aldrich	? mL chitosan 1.5 mg/mL or 2 mg/mL (in acetic acid)	1 mL of insulin 20 mg/mL	Premixed with chitosan solution	? mL TPP 2.5 mg/mL	pH 5	465 nm or 661 nm	–	77.3% ± 0.5% to 78.9% ± 0.25%	–	–	Sadhasivam et al., 2015
Chit-TPP- micro emulsion	Recombinant human insulin (Humulin R 100 IU/mL) Eli Lilly and Company,	MMWC; 75% to 85% DDA Sigma-Aldrich, United States	? mL chitosan 3 mg/mL (in acetic acid)	–	Insulin added to solution after NPs formation	? mL TPP solution 1 mg/mL	–	80.8 nm ± 7.0 nm to 401.8 nm ± 41.7 nm	+38.1 mV to +47.0 mV	–	Viability depend on concentration (XTT assay)	–	Erel et al., 2016

**Cs NPs, chitosan nanoparticles; TPP, tripolyphosphate; AE, association efficiency; CAM assay, Chick Chorioallantoic Membrane assay; XTT assay, Cell Viability Assay. Units were converted to standardization, so they can differ from the ones at the original paper. ? refers to data that could not be confirmed in the respective publication and thus remain unknown.**

Moreover, Vasiliev (2015) pointed out the importance of method harmonization and validation to chitosan analysis, such as size exclusion chromatography (SEC) to determine MW, capillary viscosimetry to check for viscosity, nuclear magnetic resonance (NMR) to define the degree of deacetylation (DD), and *Limulus amebocyte* lysate (LAL) test to verify endotoxin content.

Other authors go even deeper with respect to chitosan characterization. Even knowing that patterns of acetylation (P_A_) – random, alternating or blockwise – are linked to different polymer functionalities, such as polymer-solvent interactions (Bellich et al., 2016; Wattjes et al., 2019) and biological activity (enzyme recognition) (Weinhold et al., 2009), it is not usually taken into consideration in papers on chitosan characterization. In fact, studies have shown that chitosan with the same DD can have different solubility properties due to different patterns of distribution of its monomers *N*-glucosamine and *N*-acetyl-glucosamine (Bellich et al., 2016). Because commercially available chitosan is produced by chemical deacetylation of chitin under heterogeneous conditions (Wattjes et al., 2019), it usually results in heterogeneous products with random patterns of acetylation (Varum et al., 1991; Weinhold et al., 2009). Enzymatic deacetylation is an interesting alternative to chitosan preparation as the application of chitin deacetylases allows for a controlled process, resulting in a polysaccharide with well-defined patterns of acetylation (Tsigos et al., 2000).

Despite different opinions, the accurate determination of chitosan properties should be unavoidable (Bellich et al., 2016). MW, DD, viscosity and purity should be presented as chitosan characterization parameters. Moreover, it is known that the properties discussed above will influence Chit NP physicochemical properties such as size and zeta potential, but also determine its biological activity. It is therefore essential to define the properties of chitosan in order to assure the reproducibility of Chit NP preparation (Hua et al., 2018) and to obtain the desired biological response. Moreover, in order to follow a SbD approach, as mentioned before, it is important to classify with accuracy the physicochemical properties that determine the safety of the nanomaterial.

### Drug Encapsulation Into Chitosan/Tripolyphosphate Nanoparticles (Chit-TPP NPs): Insulin as Case-Study

Chit NPs can be prepared through numerous methods. Among them, ionotropic gelation is based on the electrostatic interactions between charged polymers and non-toxic anionic cross-linking agent species, such as citrate, sulfate, or TPP. Ionotropic gelation is performed in aqueous media, avoiding organic solvents, high temperatures, and high shear rates. Because of that, it is a safe preparation method resulting in low-toxicity NPs (Dash et al., 2011; Bugnicourt and Ladavière, 2016). In the case of Chit NPs preparation, the convenient characteristics of ionotropic gelation along with the cationic sites available all along the polymer chain of chitosan allow the interaction and encapsulation of fragile poly-anionic molecules, such as proteins and deoxyribonucleic acids (DNA), producing stable colloidal complexes (Xu and Du, 2003; Bugnicourt and Ladavière, 2016).

Chit NP production, particularly by using TPP as a crosslinker, is a generally established method and it is by far the most mentioned in the literature. Usually, the preparation of Chit-Ins NPs by ionotropic gelation consists in dissolving the polysaccharide in an aqueous acetic acid solution, while TPP is dissolved in deionized water. Then, TPP solution is added dropwise to the chitosan solution under stirring (magnetic stirring or using a high-speed homogenizer), leading to the spontaneous formation of Chit NPs (Calvo et al., 1997).

**FIGURE 2:**
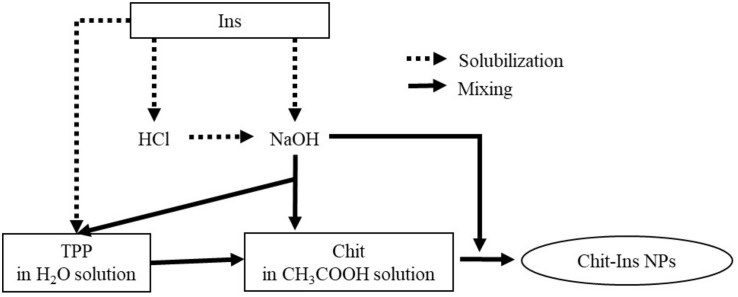
Differences on Chit-ins NPs production protocols.

There are many different protocols for insulin encapsulation into Chit NPs (Figure 2). Insulin can be pre-dissolved in diluted hydrogen chloride (HCl) solution (Abbad et al., 2015), the pH of this final solution can be adjusted with sodium hydroxide (NaOH) (Hecq et al., 2015; Li et al., 2017), or insulin can even be directly solubilized into diluted NaOH, or directly into TPP solution (Zhao et al., 2014). Then, the insulin solution is added to the chitosan solution right before or during TPP addition (Ma et al., 2005; Azevedo et al., 2011; Makhlof et al., 2011) or added after TPP addition to chitosan (Ma et al., 2002; Erel et al., 2016). Nanoparticles form spontaneously, the system stays under stirring for a while in order to stabilize the nanoparticles.

Despite similar formulation and preparation procedures, different properties of the resulting insulin-loaded chitosan nanoparticles (Chit-Ins NPs) have been reported (Ma et al., 2002), as shown in Table 1. Factors such as chitosan and TPP concentrations, pH, chitosan origin and its characteristics, rotation speed, insulin concentration, among others, greatly influence the final nanoparticle properties, thus having a serious impact on batch reproducibility and bioactivity (Ma et al., 2002; Sreekumar et al., 2018).

Note that the systems listed in Table 1 were developed mainly for the oral administration of insulin. This protein is highly susceptible to enzymatic degradation in the gastrointestinal (GI) tract, thus nanoparticles may aid to protect it from the acidic environment and enzymatic degradation, and to promote insulin absorption by using mucoadhesive polymers, such as chitosan (Makhlof et al., 2011; Al Rubeaan et al., 2016). Despite their well-known potential, Chit-TPP NPs are not stable under acidic conditions, as the protonation of the amino groups of chitosan at low pH values promotes their dissolution and successive insulin degradation, decreasing its bioavailability (Al Rubeaan et al., 2016).

In order to increase nanocarrier stability in the gastric environment recent delivery systems have been developed based on modified chitosan through conjugation, quaternization, thiolation, substitution, and grafting (Chaudhury and Das, 2011; Al Rubeaan et al., 2016). For example, permanently positively charged *N*-(2-hydroxy)propyl-3-trimethyl ammonium chitosan chloride (HTCC), increases Chit-Ins NPs stability (Hecq et al., 2015). Another derivative, thiomalyl chitosan, produces negatively charged NPs that, curiously, seem to enhance mucoadhesion and permeation, when compared to Chit NPs. This system is also suggested to inhibit insulin degradation due to its protease inhibitory effect (Rekha and Sharma, 2015).

Moreover, hydroxypropyl methylcellulose phthalate used as crosslinker (instead of TPP) in Chit-Ins NPs preparation also proved to increase NP stability and, additionally, to improve intestinal mucoadhesion and penetration (Makhlof et al., 2011). Finally, another interesting approach is an emulsion-based delivery system, where Chit-Ins NPs were suspended in a microemulsion, successfully protecting insulin under gastric conditions and reducing blood glucose levels for 8 h after oral administration (Erel et al., 2016).

As can be extracted from Table 1, depending on the preparation method, reported NP size may range between 112 and more than 400 nm. Zeta potential, when measured, was also highly variable with values ranging between 20 and 40 mV. Even more variable was the encapsulation efficiency for insulin reported, with values ranging from as little as 2% to almost 90%. Overall it can be said that generally not all relevant data on the materials and methods used were reported, rendering the selection of the optimal preparation method from the literature difficult.

### Chitosan as an Immunostimulant: An Additional Source of Disagreement

As mentioned before, chitosan is known for its immunostimulatory activity. Because of that, the polysaccharide has been extensively studied and reviewed as an adjuvant and/or as a delivery system for vaccines (Van der Lubben et al., 2001a; Ghendon et al., 2009; Esmaeili et al., 2010; Mehrabi et al., 2018).

Establishing the physicochemical properties that are correlated with chitosan immune stimulation is important to define Chit NPs activity in view of a SbD approach. However, as for other data available for chitosan, reports on its immunomodulation activity are contradictory. Some publications claim that chitosan is not able to stimulate antibody production (de Geus et al., 2011), while other studies confirm that chitosan can only induce immunostimulation due to the synergic effect between the components of the chitosan formulation and the antigen (Seferian and Martinez, 2000; Bivas-Benita et al., 2004). In addition, many articles claim the obvious adjuvant potential of the polysaccharide (Nishimura et al., 1985; Zaharoff et al., 2007; Ghendon et al., 2009; Esmaeili et al., 2010; Dzung et al., 2011; Vasiliev, 2015; El Temsahy et al., 2016).

The adjuvant activity of chitosan was first attributed to its mucoadhesive properties, which prolong the residence time of the loaded antigen at mucosal sites. This, in turn, increases antigenic uptake (Illum, 1998; Alpar et al., 2005) and improves immunological response via transmucosal routes (Illum, 1998): nasal (Van der Lubben et al., 2001b; Esmaeili et al., 2010), pulmonary (Esmaeili et al., 2010), and oral (Van der Lubben et al., 2001b; Borges et al., 2006, 2007; Esmaeili et al., 2010). Furthermore, the physical association of chitosan with an antigen (Calvo et al., 1997; Seferian and Martinez, 2000) and its slow release are very important to the overall adjuvant activity of the biopolymer (Calvo et al., 1997).

Other authors explored the potential of chitosan immune stimulation through the parenteral route (Borges et al., 2008), based on preliminary data that attributed adjuvant activity to chitin derivatives, including chitosan. These biopolymers increased immune response in guinea pigs after immunization applied to their footpads (Nishimura et al., 1985). Zaharoff et al. (2007) vaccinated mice with β-galactosidase dissolved in a viscous chitosan solution. The adjuvant activity was attributed to the combination of an antigen depot with the stimulation of both humoral and cell-mediated immune responses (Zaharoff et al., 2007). Correspondingly, Ghendon et al. (2009) explored the properties of chitosan as an adjuvant for inactivated influenza vaccines, showing that the polysaccharide induced the production of high titers of antibodies against the antigen and increased cytotoxic activity of NK-cells. Furthermore, Chit NPs are known to induce mixed Th1/Th2 responses with a great variability of antigens. An increase of interferon-γ (IFN-γ) and IgG2a is characteristic for a Th1 response, while the Th2 pathway is elicited by IL-4 and IgG1 production (Zaharoff et al., 2007; Borges et al., 2008). Additionally, Chit NPs interact with antigen-presenting cells (APCs), such as macrophages, and induce CD4^+^ T cell proliferation (Zaharoff et al., 2007). In case of mucosal administration, an increased production of sIgA has been shown (Vila et al., 2004; Borges et al., 2007).

Recently, Chit NPs prepared by ionotropic gelation have been tested as an adjuvant in several vaccine systems (Vila et al., 2004; Danesh-Bahreini et al., 2011; Dzung et al., 2011; El Temsahy et al., 2016). For example, El Temsahy et al. (2016) produced *Toxoplasma* lysate vaccines by encapsulating virulent RH and avirulent Me49 *Toxoplasma* strains into Chit NPs, while Danesh-Bahreini et al. (2011) applied the Chit-TPP system to develop a leishmaniasis vaccine. In the first example, the *Toxoplasma* lysate vaccines were injected by the intraperitoneal route into mice, stimulating both humoral and cellular immune responses (El Temsahy et al., 2016). Furthermore, the Chit-TPP-antigen system was shown to be as effective as Freund’s incomplete adjuvant (FIA) in enhancing the efficacy of *Toxoplasma* vaccine (El Temsahy et al., 2016). The reported data are in agreement with other studies comparing the polysaccharide with commonly used adjuvants, FIA and aluminum hydroxide, demonstrating the biopolymer to be equipotent to those adjuvants (Zaharoff et al., 2007; Dzung et al., 2011). Chit NPs where also loaded with *Leishmania* superoxide dismutase (SODB1), and injected into BALB/c mice, eliciting both IgG2a1 and IgG1 production (Danesh-Bahreini et al., 2011). Therefore, chitosan is an alternative to traditional adjuvants applied in vaccine development (Zaharoff et al., 2007; El Temsahy et al., 2016).

In general, immune responses depend on the system’s physicochemical characteristics, properties and dose of antigen (Amidi et al., 2010). Furthermore, polysaccharide features appear to influence the elicited response. Chitosan from different sources and suppliers, of different DD (Nishimura et al., 1985; Scherliess et al., 2013) and MW (Ghendon et al., 2009; Dzung et al., 2011; Scherliess et al., 2013) have been used to explore its immunostimulant activity. Nishimura et al. (1985) observed a correlation between the immunological activity and chitosan DD, in which 70% DD was the optimal value, whereas 30% DD resulted in lower adjuvanticity. This appears to be in agreement with data showing that positively charged particles are associated with increased immunogenicity (Foged et al., 2005). However, recent reports also showed that chitosan with 76% DD elicited higher immune responses than 81% DD chitosan (Scherliess et al., 2013).

Data is also contradictory with respect to the influence of MW on chitosan immunostimulant activity. While some authors claim that LMW chitosan (10 kDa) is more effective in immune system stimulation than HMW chitosan (300 kDa) (Ghendon et al., 2009), others show that MW around 300 kDa has a greater effect than LMW chitosan (Dzung et al., 2011). Moreover, another paper stated that MW had no significant impact on Chit NPs stimulated immune response (Vila et al., 2004). Note that the last classification of LMW and HMW was based on Ghendon et al. (2009).

The contradictory information suggests that the chitosan formulation can also affect its adjuvant action (Scherliess et al., 2013). In case of chitosan particulate systems, the preparation technique has a direct influence on the particle size, which also influences the triggered immune pathway (Bueter et al., 2011; Scherliess et al., 2013; Soares and Borges, 2018). Note that the particle size also depends on chitosan MW and DD (Scherliess et al., 2013). Moreover, the antigen release pattern from the chitosan system and the injection site seem to affect the immune response, as well (Vila et al., 2004; Scherliess et al., 2013).

Furthermore, there is a lack of information on the biopolymer purity, such as the presence of endotoxins, LPS, proteins, nucleic acids and heavy metals, which can have an important influence on the immune response elicited. As a consequence, it has been proposed that the adjuvant activity attributed to chitosan can be related to its impurities and not to the polymer itself (Vasiliev, 2015).

In the end, it is not clear which factor is responsible for the differences in immune responses elicited by the biopolymer. There is most probably an interaction between all the properties mentioned before affecting chitosan adjuvanticity (Scherliess et al., 2013).

### Undesired Adjuvanticity of Chit: Potential Immunotoxicity of Chit-Ins NPs

The adjuvant activity of chitosan has been studied for the purpose of vaccine formulation. That means that the active pharmaceutical ingredient (API) encapsulated is already known to have immunogenic properties, whether the antigen is highly or poorly immunogenic. The great majority of Chit-TPP systems loaded with insulin are studied as an alternative to the subcutaneous administration of insulin. Thus, immunogenic studies are not usually a concern as shown in Table 1, which illustrates the lack of information on the immunotoxicological and immunopharmacological profile of Chit-Ins NPs.

Note that mucosal delivery routes—oral, nasal, etc.—studied for insulin administration generally imply absorption through a mucosal surface, where chitosan has also been widely applied as a vaccine adjuvant (Illum, 1998; Van der Lubben et al., 2001b). Insulin is indeed poorly immunogenic (Fineberg et al., 2007). Its formulations for subcutaneous administration have been developed and improved, indicating rare severe immunological complications. Actually, less than 0.1% of recipients experience insulin resistance due to immune reactions (Fineberg et al., 2007). However, insulin resistance due to Chit-Ins NPs administration cannot be totally excluded in the absence of in-depth studies.

Chit NPs adhere to the mucosa and transiently open intercellular tight junctions. Due to the pH variation, these NPs become less stable and disintegrate releasing the insulin, which is absorbed through the paracellular pathway into the systemic circulation (Borchard et al., 1996; Sung et al., 2012). In reality, other transport pathways can be involved after oral administration of Chit-Ins NPs (Abbad et al., 2015), such as transcytosis through enterocytes, receptor-mediated transcytosis, and transcellular absorption by M cells in the Peyer’s patches. As part of the gut associated lymphoid tissue (GALT), Peyer’s patches have an important role in eliciting immune responses against oral antigens, as reviewed elsewhere (Soares and Borges, 2018). However, since absorption studies do not use models that include enterocytes, goblet, and M cells simultaneously, the insulin absorption pathway is still unknown (Abbad et al., 2015). Furthermore, these studies showed NP uptake by epithelial cells, but did not prove their transport across those cells. Thus, there is a risk of intercellular degradation of the NPs (Amidi et al., 2010; Hu and Luo, 2018).

Depending on the route of administration, Chit-Ins NPs can be taken up and processed by APCs, or transported into lymphatic tissues, triggering a local and/or systemic immune response against the protein (Amidi et al., 2010; Soares and Borges, 2018). Furthermore, it should be kept in mind that the repeated administration of the formulation increases the potential risk of antibody formation against insulin (Jiskoot et al., 2009).

### The Hurdles of Protein Delivery by Chit-NPs

Even though there is plenty of information on chitosan in the literature, there is also a huge gap with regard to chitosan standardization, making it difficult to relate its characteristics with the outcomes reported (Vasiliev, 2015) and to establish guidelines for SbD implementation. Note that polymer composition is a requirement of the assay cascade for nanomedicines elaborated by both US NCL and EU-NCL (European Nanomedicine Characterization Laboratory, 2019b; Nanotechnology Characterization Lab, 2019), thus the complete characterization of chitosan is revealed to be the greatest need and challenge of all.

The FDA Department Guidance for Industry “Drug Products, Including Biological Products that Contain Nanomaterials” requires the full description of nanomaterial composition, based on their functionality and intended use (U. S. Food and Drug Administration, 2017). Furthermore, the FDA guidance states that the nanomaterial critical quality attributes (CQAs) should be determined as early as possible, considering their functions and potential impact on the final product performance (quality, safety, and efficacy). Moreover, risk assessment should be applied linking the structure-function relationship of the nanomaterial to attributes that need to be examined and controlled in case of manufacturing changes – for example, the source and supplier of chitosan for NP production (U. S. Food and Drug Administration, 2017). Scarce good laboratory practice (GLP) conditions and questions regarding the validity and reproducibility of the scientific results are obstacles to collaboration with pharmaceutical industry and approval by regulatory authorities (Rosenblum et al., 2018). For example, clinical translation relies on a consistent and reproducible product (Anselmo and Mitragotri, 2016). As far as chitosan is concerned, contradictory information available in the literature on chitosan-biological activity correlation may be a potential source of problems during the drug approval process.

The risk assessment approach should also be applied to evaluate possible adverse immune responses that may be associated with nanomaterial administration, affecting both safety and efficacy. Biological products with a nanomaterial component may have a different immunogenic profile compared to the biological substance alone, which may apply to Chit-Ins NPs (U. S. Food and Drug Administration, 2017).

As reviewed elsewhere (Jiskoot et al., 2009), the particulate character of drug delivery systems makes them predisposed to be recognized as foreign by immune cells and the complement system. In general, the elicited immune response depends on the route and frequency of administration. Moreover, in case of Chit-Ins NPs the potential immune response will also depend on chitosan characteristics and its source, on the properties of the nanocarrier (size, surface charge, polydispersity, etc.), and on the insulin employed. Often recombinant human insulin is applied, which usually does not stimulate immune responses. However, the immunogenicity risk of frequent administration of Chit-Ins NPs is unknown, as chitosan is known to have adjuvant properties, and recombinant human therapeutic proteins are also known to trigger antibody production after chronic treatment (Hermeling et al., 2004). Chitosan systems stimulate both cellular and humoral responses. Therefore, studies should be carried out to detect anti-insulin IgG1 and IgG2a production after Chit-Ins NPs administration. Screening of cytokine production, such as IL-4 and IFN-γ, and detection of IgA, in the case of mucosal administration, would also be of interest.

In the end, the potential problems regarding Chit-Ins NP administration can be analyzed from a larger scope. The application of Chit NPs to protein delivery, in general, should take into account chitosan characteristics and the potential triggering of an immune response. These must be taken into consideration when examining the human health risks of a formulation in the framework of a SbD approach, especially when it is not desirable to stimulate the immune system.

## Conclusion

This review shows that the characterization of chitosan is frequently missing in scientific reports, which complicates the translation into a SbD driven approach. Since the term chitosan is applied to a large group of polymers, the biological effects can be different and dependent on the degree of deacetylation and molecular weight of the polymer used on the study. This fact may explain, at least in part, the contradictory biological effects of chitosan reported in literature. Moreover, the purity of the polymers is not always mentioned, and the observed effects may be influenced by the presence of contaminants and impurities. Additionally, a similar situation can be observed with Chit NPs. Several protocols can be found in literature for insulin encapsulation into Chit NPs, however, in view of the lack of complete information given, it is difficult to reproduce them. Protocols also differ, which is an additional problem for data analysis and its comparison.

Furthermore, even though the immunostimulatory effect of chitosan systems has been well reported in the vaccine delivery field, the undesirable potential immune stimulation of those nanocarriers has been given less attention.

The data presented in this report illustrate the challenges encountered when implementing the SbD concept to polymeric drugs based on chitosan. The SbD approach defined by GoNanoBioMat establishes an early risk identification through material design and characterization. However, as it is shown in this report, the correlation between chitosan’s physicochemical properties and its activity is far from being established. Consequently, it is also difficult to correlate Chit NP characteristics with the efficacy of the final drug product. Moreover, the potential hazard, namely, the eliciting of an unwanted immune activity, is also difficult to predict.

The full understanding of the composition of the nanoformulation is a critical point, thus a lack of knowledge in this field may explain why the number of approved drugs with chitosan as excipient is limited. Harmonization and validation of chitosan analysis will enable comparison between future studies. By developing these studies, it will be possible to establish the characteristics of different types of chitosan nanoparticles, establish a correlation between chitosan properties and its immunostimulant activity and, finally, to establish a guideline to select the most appropriate chitosan according to its purpose, allowing a safe-by-design approach.

## Author Contributions

CM drafted the manuscript, was responsible for the acquisition, analysis, and interpretation of the data for the work. CS, MS, and OB provided critical revision and redrafted the manuscript. GB provided critical revision, redrafted the manuscript, and gave approval for publication of the content.

## Conflict of Interest

The authors declare that the research was conducted in the absence of any commercial or financial relationships that could be construed as a potential conflict of interest.
